# A score test for comparing cross-sectional survival data with a fraction of non-susceptible patients and its application in clinical immunology

**DOI:** 10.1371/journal.pone.0179896

**Published:** 2017-06-30

**Authors:** Sarah Flora Jonas, Cyprien Mbogning, Signe Hässler, Philippe Broët

**Affiliations:** 1 CESP, Université Paris-Sud, UVSQ, INSERM, University Paris-Saclay, Villejuif, France; 2 Abirisk consortium WP4, 14-16 Avenue Paul-Vaillant Couturier, Villejuif, France; 3 Assistance Publique - Hôpitaux de Paris, Hôpital Paul Brousse, 14-16 Avenue Paul-Vaillant Couturier, Villejuif, France; University of North Carolina at Chapel Hill, UNITED STATES

## Abstract

**Objectives:**

In cross-sectional studies of time-to-event data collected by patient examinations at a single random point in time, a fraction of them will not experience the event regardless of the length of the follow-up time. This is the case in clinical immunology studies that include a mixed population, with both immune-reactive and immune-tolerant (or non-susceptible) patients. In these cases, classical tests of current status data may perform poorly. New methods for testing these data are needed.

**Methods:**

In the two-sample comparison setting, we propose a score test for testing the null hypothesis that survival does not differ in either the non-susceptible fraction or the time-to-event distribution among the susceptible fraction.

**Results:**

In a wide range of scenarios, simulation results show interesting improvements in power for the proposed score test compared to the logrank-type test in most of the configurations we investigated. In a cross-sectional study of drug immunogenicity among treated multiple sclerosis patients, the proposed score test reveals that gender is associated with the immunogenicity of interferon.

## Introduction

Biopharmaceutical products (BP) are currently one of the fastest growing groups of drugs used in clinical immunology to treat patients with immune-based disorders, such as multiple sclerosis, rheumatoid arthritis, and inflammatory bowel diseases. These products, however, may lead to the development of antibodies (either binding or neutralizing) directed against the drug—known as anti-drug antibodies or ADAs—with treatment failures as a potential clinical consequence [1]. The prediction of ADA occurrence is a challenging problem today. Limited resources lead most studies conducted by clinical laboratories searching for the risk factors of ADAs to rely on cross-sectional sampling in which patients receiving BP are tested for ADA status (positive/negative) at a single random point in time. ADA status, however, is an active process that depends upon the dynamics of ADA production by (T-cell dependent) B-lymphocyte clones. Specific methodologies for time-to-event outcomes must be used to analyze such data. In addition, no information is available for individual patients between the first drug administration and the monitoring time point, which means that the only information available about the time-to-occurrence of ADA is whether it exceeds the random monitoring time point. This special kind of data, which is known as current status data (or case I interval-censored data), requires particular methods that differ substantially from those used for classical right-censored data [2, 3].

Several k-sample tests have been proposed to compare time-to-event distributions of current status data. They rely on efficient score statistics that can be expressed as either rank tests or weighted logrank-type tests (for a review, see [3]). These statistics allow investigators to test for equality of hazard functions against constant or non-constant (over time) hazard ratio alternatives [3, 4]. In general, the weighted logrank-type tests use weight functions that are either motivated by the expected deviation from the null hypothesis or model-based with some optimal properties for a particular family of alternatives (e.g. the *G*^*ρ*^ extended family of Harrington-Fleming survival distributions as seen in [5, 6]).

However, all these statistics rely on the assumption of so-called proper survival distributions. Broadly speaking, it is assumed that if the follow-up time is long enough, all patients will eventually experience the event of interest. This is obviously not the case in an investigation of BP immunogenicity; instead, we expect that a fraction of the patients receiving BP are immune-tolerant (non-susceptible) to the drugs and will not experience ADA at all during the long-term follow-up. The other patients are immune-reactive (susceptible) to ADA, and their time-to-ADA detection depends on the dynamics of ADA clonal production by B lymphocytes. Thus, the bioclinical factors studied may be associated with differences either in the proportion of immune-tolerant patients or in the distribution of the time-to-ADA occurrence among immune-reactive patients. For classical right-censored data, this problem of a mixed population has been tackled mainly from two different frameworks, one relying on two-component mixture cure models and the other on bounded cumulative hazard models [7].

The first approach considers that the study population is a mixture of two groups of patients: non-susceptible and susceptible. This formulation has led to proposals for various parametric and non-parametric models [7–10]. The second approach, called a promotion time cure model [11], assumes that the observed time-to-event is the first of some latent event time and has interesting mechanistic interpretations in various biological fields, such as oncology. Such a first-activation scheme with a Poisson process leads to the bounded cumulative hazard model introduced by Yakovlev and Tsodikov [12–14].

Although many estimates and testing methods for these cure models have been proposed, few have been implemented in classical survival analysis software (such as R or SAS), and none is designed to cope with interval-censored data. The latter fact explains why, at best, investigators today use the interval-censored tests that have previously been implemented, but ignore the mixed population issue.

We recently faced this methodological problem in analyzing the immunogenicity of interferon among multiple sclerosis patients. It prompted us to develop a procedure for testing the null hypothesis of equality of the two survival functions with a fraction of non-susceptible patients.

The main purpose of this work is thus to provide a simple test able to detect survival differences, for use by practitioners with cross-sectional data from a mixed population.

In the Methods section, we first introduce a semi-parametric improper survival model that allows us to describe changes in the non-susceptible fraction and/or in the survival distribution in the susceptible fraction. We then present a score test for the two-sample problem to test the null hypothesis that the variable under consideration has no effect on either the proportion of susceptible patients or the time-to-event distribution. This test relies on the components of the score statistic obtained under the nu ll hypothesis and can be re-expressed as a vector of linear rank statistics. In the Results section, the Simulation study subsection reports the results of simulation experiments performed to study the power properties of the proposed test and compare them with those of the classical logrank test for current status data. In the second Results subsection, we use the proposed test to analyze the predictive effect of gender on the occurrence of ADA. In the last section, the Discussion, we review the advantages and limitations of the proposed test and its potential extensions. We also give some advice for its practical use.

## Methods

### Notations

Let the continuous random variables *T* and *C* represent the unobservable failure and monitoring time, respectively. Let *f*(*t*) denote the probability density function, and *S*(*t*) (*resp*. $\bar{S}\left(t\right)$) the survival function (*resp*. cumulative distribution function) of *T*. The hazard function (or the instantaneous event rate) of *T* is *h*(*t*) with *h*(*t*) = *f*(*t*)/*S*(*t*).

For current status data, we observe only whether the event of interest occurred before some single random monitoring time. Here, for each patient *i* (*i* = 1, …, *n*), *Z*_*i*_ is a binary variable that indicates group membership (*Z*_*i*_ = 0 or 1, with 0 the reference group). Thus, (*C*_*i*_, $\delta_{i}=1_{\left(C_{i}\geq T_{i}\right)}$, *Z*_*i*_) comprise the observed data. If an event occurred, we know that *T*_*i*_ belongs to [0, *C*_*i*_]; otherwise *T*_*i*_ belongs to [*C*_*i*_, ∞_+_[. Here, we assume that the censoring and the failure times are independent. We also assume that the censoring times are independent and identically distributed random variables for all subjects.

### Improper survival model

#### Rationale for considering a bounded cumulative hazard model

The biological mechanisms of ADA immunogenicity as well as pragmatic statistical considerations led us to consider a bounded cumulative model. The main idea is to model the distribution of the ADA detection time through a simplified mechanistic immunological model whereby each individual is potentially able to produce ADAs that arise from the activation of latent (or unobservable) BP-specific (T-dependent) B-cell clones. At the cellular level, each one of these clones can emerge and become an immunocompetent ADA-producing clone. Once a BP-specific B-cell clone is activated, its production leads sooner or later to ADA detection. ADA status becomes positive for the first time as soon as any of these immunocompetent BP-specific B-cell clones produces sufficient antibodies to reach the detection threshold.

From a statistical point of view, assuming relevant probability distributions for both the number of latent B-cell clones and the time-to-ADA detection, we can deduce the marginal (or population) survival distribution of this time to detection. In the spirit of the stochastic models developed in the seminal work of Yakovlev and Tsodikov [12], we assume that the number of B-cell clones is distributed as a Poisson distribution and that the clones are independent. This leads to the bounded cumulative model presented just below.

#### Survival model

In this work, we consider the following semi-parametric improper survival model such that for patient *i* we have:

$$S\left(t|Z_{i}=z\right)=S_{z}\left(t\right)={\text{e}}^{-\theta {\text{e}}^{\alpha z}[1-\exp(-\Lambda \left(t\right){\text{e}}^{\beta z})]}$$ (1)

where Λ(*t*) is an unspecified increasing positive function from zero to infinity.

For the hazard functions, *h*_0_(*t*) and *h*_1_(*t*):

$$h_{1}(t)=h_{0}(t)\;{\text{e}}^{\alpha +\beta }{\text{e}}^{-\Lambda (t)\left({\text{e}}^{\beta }-1\right)}.$$

The survival function *S*_*z*_(*t*) is improper in the sense that lim_*t*→+∞_ *S*_*z*_(*t*) > 0. Its limiting value is called the tail defect (sometimes referred as the plateau) and here equals e^−*θ*e^*αz*^^. In our setting, it represents the probability of being immune-tolerant. Changes in the immune-tolerant fraction and in the time-to-event distribution (here the dynamics of ADA production) are modeled through the parameters of interest *α* and *β*. Thus when *α* = 0, the two groups have the same plateau (proportion of non-susceptible patients). And when *β* = 0, Model (1) is a proportional hazard model with a relative risk constant over time with a different plateau value.

Under Model (1), the simplified log-likelihood for the *n* observed current status data is:

$$l_{n}\left(\alpha ,\beta ,\theta ,\Lambda \left(.\right)\right)=\sum_{i=1}^{n}\delta_{i}\;\{\log(1-{\text{e}}^{-\theta {\text{e}}^{\alpha z_{i}}[1-\exp(-\Lambda (c_{i}){\text{e}}^{\beta z_{i}})]})\}\;-\;(1-\delta_{i})\;\{\theta {\text{e}}^{\alpha z_{i}}\;[1-\exp(-\Lambda (c_{i}){\text{e}}^{\beta z_{i}})]\}.$$

### Score test for *H*_0_ : *α* = *β* = 0

The null hypothesis *H*_0_ : *α* = *β* = 0 to be tested is the equality of the two improper survival distributions, that is, that group membership has no effect on either time-to-event distribution or the immune-tolerant fraction. Under the null hypothesis *H*_0_, the score vector has the following components *U* = (*U*_*α*_, *U*_*β*_) where:

$$U_{\alpha }=\frac{\partial l_{n}}{\partial \alpha }{|}_{\alpha =\beta =0}=\sum_{i=1}^{n}\delta_{i}\;\{\frac{{\text{e}}^{-\theta \;[1-\exp(-\Lambda (c_{i}))]}\theta \;[1-\exp(-\Lambda (c_{i}))]}{1-{\text{e}}^{-\theta [1-\exp(-\Lambda (c_{i}))]}}z_{i}\}\;-\;(1-\delta_{i})\;\{\theta \;[1-\exp(-\Lambda (c_{i}))]z_{i}\}.$$

Thus,

$$\begin{array}{ccc} U_{\alpha } & = & \sum_{i=1}^{n}\;z_{i}\;[\frac{\theta \;[1-\exp(-\Lambda (c_{i}))]}{1-{\text{e}}^{-\theta [1-\exp(-\Lambda (c_{i}))]}}]\;\{\delta_{i}-1+{\text{e}}^{-\theta [1-\exp(-\Lambda (c_{i}))]}\},\\ & = & \sum_{i=1}^{n}\;z_{i}w_{\alpha ,i}. \end{array}$$

And,

$$U_{\beta }=\frac{\partial l_{n}}{\partial \beta }{|}_{\alpha =\beta =0}=\sum_{i=1}^{n}\delta_{i}\;\{\frac{{\text{e}}^{-\theta [1-\exp(-\Lambda (c_{i}))]}\theta \Lambda (c_{i}){\text{e}}^{-\Lambda (c_{i})}}{1-{\text{e}}^{-\theta [1-\exp(-\Lambda (c_{i}))]}}z_{i}\}\;-\;(1-\delta_{i})\;\{\theta \Lambda (c_{i}){\text{e}}^{-\Lambda (c_{i})}z_{i}\}.$$

Thus,

$$\begin{array}{ccc} U_{\beta } & = & \sum_{i=1}^{n}\;z_{i}\;[\frac{\theta \Lambda (c_{i}){\text{e}}^{-\Lambda (c_{i})}}{1-{\text{e}}^{-\theta [1-\exp(-\Lambda (c_{i}))]}}]\;\{\delta_{i}-1+{\text{e}}^{-\theta [1-\exp(-\Lambda (c_{i}))]}\},\\ & = & \sum_{i=1}^{n}\;z_{i}w_{\beta ,i}. \end{array}$$

As shown from the preceding two formulas, these score statistics can be rewritten as linear rank statistics with the following ranking-like functions *w*_*α*,*i*_ and *w*_*β*,*i*_ for subject *i*, which depend upon the cumulative distribution function under the null hypothesis.

$$\begin{array}{c} w_{\alpha ,i}(c_{i})=[\frac{-\log(1-{\bar{S}}_{H_{0}}(c_{i}))}{{\bar{S}}_{H_{0}}(c_{i})}]\;\{\delta_{i}-{\bar{S}}_{H_{0}}(c_{i})\}, \end{array}$$

$$\begin{array}{c} w_{\beta ,i}(c_{i})=[\frac{-\theta \log(1+\frac{\log(1-{\bar{S}}_{H_{0}}(c_{i}))}{\theta })\;[1+\frac{\log(1-{\bar{S}}_{H_{0}}(c_{i}))}{\theta }]}{{\bar{S}}_{H_{0}}(c_{i})}]\;\{\delta_{i}-{\bar{S}}_{H_{0}}(c_{i})\}, \end{array}$$

where ${\bar{S}}_{H_{0}}\left(.\right)$ is the cumulative distribution function under the null hypothesis *H*_0_.

In practice, we replace ${\bar{S}}_{H_{0}}\left(t\right)$ and *θ* by ${\hat{\bar{S}}}_{H_{0}}\left(t\right)$ and $\hat{\theta }$ applying the following ad hoc approach. For interval-censored data, a non-parametric maximum likelihood estimator (NPMLE) for the cumulative distribution function under the null hypothesis ${\hat{\bar{S}}}_{H_{0}}\left(t\right)$ can be obtained by running the classical pooled-adjacent-violators algorithm on the full dataset [15]. This estimator uses the estimated jumps (probability mass) occurring over the so-called innermost intervals [16]. In our setting with an improper survival distribution, we arbitrarily consider that for the last innermost interval (the upper limit of which is infinite), the jump is set to zero. Thus, under the null hypothesis, an estimator of *θ* is obtained by $\hat{\theta }=-log\left({\hat{\bar{S}}}_{H_{0}}\left(\infty \right)\right)$ where ${\hat{\bar{S}}}_{H_{0}}\left(\infty \right)$ is the estimate of the tail defect and its value is the difference between the last estimate of 1 and ${\hat{\bar{S}}}_{H_{0}}\left(t\right)$. Moreover, when ${\bar{S}}_{H_{0}}\left(.\right)=0$, by convention ${\bar{S}}_{H_{0}}\left(.\right)=\min_{{\bar{S}}_{H_{0}>0}}\left({\bar{S}}_{H_{0}}\left(.\right)\right)$.

This approach relies on the hypothesis that the censoring mechanism verifies a condition of sufficient follow-up, that is, that the susceptible subjects will experience the event within the follow-up period [8]. In practice, it implies that we should allow a period of observation that is long enough to detect the presence of immune-tolerant individuals in the study population (i.e. the last interval should be event-free).

Since the statistics *U*_*α*_ and *U*_*β*_ can be expressed as linear rank statistics, we can obtain their permutational variances and the covariance under the null hypothesis (see [2, 17]):

$$V_{U_{\alpha }}=\frac{1}{n-1}\;[\sum_{i=1}^{n}\;{\left(w_{\alpha i}-{\bar{w}}_{\alpha }\right)}^{2}\;\sum_{i=1}^{n}\;{\left(z_{i}-\bar{z}\right)}^{2}],$$

$$V_{U_{\beta }}=\frac{1}{n-1}\;[\sum_{i=1}^{n}\;{\left(w_{\beta i}-{\bar{w}}_{\beta }\right)}^{2}\;\sum_{i=1}^{n}\;{\left(z_{i}-\bar{z}\right)}^{2}],$$

$$\text{cov}\left(U_{\alpha },U_{\beta }\right)=\frac{1}{n-1}\;[\sum_{i=1}^{n}\;(w_{\alpha i}-{\bar{w}}_{\alpha })\;(w_{\beta i}-{\bar{w}}_{\beta })\;\sum_{i=1}^{n}\;{\left(z_{i}-\bar{z}\right)}^{2}],$$

with $\bar{w_{.}}=\frac{1}{n}\;\sum_{i=1}^{n}\;w_{.i}$ and $\bar{z}=\frac{1}{n}\;\sum_{i=1}^{n}\;z_{i}$.

Finally, the proposed test statistic of *H*_0_ is given by:

$$T_{H_{0}}=(U_{\alpha },U_{\beta })\;V^{-1}\;(\begin{array}{c} U_{\alpha }\\ U_{\beta } \end{array})$$

where *V* is the matrix of variance-covariance obtained with the preceding formulas.

Under the null hypothesis, the statistic *T*_*H*_0__ is asymptotically distributed as a chi-square with two degrees of freedom.

## Results

### Simulation study

#### Protocol

To examine the properties of this test, we conducted Monte-Carlo simulations with data generated under either a bounded cumulative hazard model or a two-component mixture cure model. We compared the proposed score test to the logrank-type test for interval-censored data [18], which is the test statistic used most often for interval-censored data. Both test the same null hypothesis of equality of the two survival distributions. Note that the logrank-type test supposes a mis-specified model in which the cure fraction is not considered. We review the formula for the logrank-type test in the appendix. In this simulation study, we investigated the impact of various percentages of censoring rates, covariate imbalances and restricted follow-ups.

Survival times were generated according to the two models described below.

The first was a bounded cumulative model such that: *S*(*t* ∣ *Z* = *z*) = e^−*θ*e^*αz*^[1 − exp(−*t*e^*βz*^)]^. The other was a two-component mixture model such that: *S*(*t* ∣ *Z* = *z*) = e^−*θ*e^*αz*^^ + (1 − e^−*θe*^*αz*^^) e^−*t*e^*βz*^^.

The variable *Z* was generated according to a Bernoulli distribution of parameter *ξ*. Unless otherwise stated, *ξ* = 0.5. The censoring (monitoring) times, *C*, were independently generated from an exponential distribution with rate parameter λ_*C*_; its value was chosen according to the desired percentage of censored susceptible observations.

The total number of subjects *n*_tot_ was set at 400.

The following configurations were considered: *β* varied from −3.2 to 3.2 with a pitch of 0.4, and *α* varied from −0.5 to 0.5 with a pitch of 0.25. The plateau values for the reference group, *τ*_0_ = *S*_*Z* = 0_(∞) = *e*^−*θ*^, were set at 0.3, 0.5, and 0.7. We indicated for each value of *α* the plateau value for the group *Z* = 1 such that $\tau_{1}=\tau_{0}^{\text{exp}(\alpha )}$. The censoring rates (*p*) used were 20% and 40%. Here, *p* refers only to the percentage of censored observations among the susceptible subjects. Thus, the total percentage of censored subjects for the reference group is equal to *τ*_0_ + *p*(1 − *τ*_0_). The results are presented in Tables 1–6 and in Tables A-F in S1 File.

**Table 1:** Bounded cumulative hazard model, exponential censoring, *τ*_0_ = 30%, *p* = 20%, *n*_tot_ = 400, *ξ* = 0.5.

*β*	-3.2	-2.8	-2.4	-2.0	-1.6	-1.2	-0.8	-0.4	0	0.4	0.8	1.2	1.6	2.0	2.4	2.8	3.2
*α*																	
-0.5	100.0	100.0	99.8	99.8	99.6	98.9	97.8	94.7	89.8	84.4	78.8	74.3	71.1	68.2	69.9	69.1	68.0
*τ*_1_ = 0.48	(0.0)	(0.1)	(-0.1)	(-0.1)	(-0.2)	(-0.4)	(-0.9)	(-2.5)	(-4.5)	(-6.2)	(-5.7)	(-3.3)	(0.7)	(4.2)	(9.5)	(10.6)	(13.5)
-0.25	99.9	99.9	99.8	99.4	98.1	94.0	81.5	58.3	34.0	19.2	19.0	27.6	39.8	54.8	65.0	72.6	77.6
*τ*_1_ = 0.39	(0.1)	(0.1)	(0.3)	(0.4)	(0.8)	(0.7)	(-1.3)	(-7.5)	(-9.6)	(-3.1)	(8.9)	(22.4)	(33.9)	(44.8)	(50.8)	(52.1)	(53.1)
0	99.9	99.8	99.7	98.6	93.6	77.5	43.2	13.9	5.1	14.1	45.0	78.1	93.2	98.7	99.6	99.9	99.9
	(0.4)	(0.7)	(1.4)	(3.5)	(7.7)	(11.6)	(6.1)	(1.0)		(0.9)	(7.9)	(11.9)	(8.4)	(4.3)	(1.7)	(0.6)	(0.3)
0.25	100.0	99.9	99.0	95.7	83.0	51.1	19.6	11.9	34.0	73.6	96.2	99.9	100.0	100.0	100.0	100.0	100.0
*τ*_1_ = 0.21	(1.2)	(2.6)	(6.8)	(15.3)	(28.4)	(28.3)	(14.2)	(1.1)	(-9.1)	(-6.4)	(-0.9)	(0.0)	(0.0)	(0.0)	(0.0)	(0.0)	(0.0)
0.5	99.9	99.6	97.7	89.0	66.6	37.3	29.3	53.6	88.6	99.4	100.0	100.0	100.0	100.0	100.0	100.0	100.0
*τ*_1_ = 0.14	(2.7)	(7.7)	(19.3)	(40.1)	(47.4)	(32.1)	(11.0)	(-8.2)	(-5.1)	(-0.3)	(0.0)	(0.0)	(0.0)	(0.0)	(0.0)	(0.0)	(0.0)

$\tau_{1}=\tau_{Z=1}=\tau_{0}^{\text{exp}(\alpha )}$/ *τ*_0_ = *τ*_*Z* = 0_ = 0.3

**Table 2:** Bounded cumulative hazard model, exponential censoring, *τ*_0_ = 50%, *p* = 20%, *n*_tot_ = 400, *ξ* = 0.5.

*β*	-3.2	-2.8	-2.4	-2.0	-1.6	-1.2	-0.8	-0.4	0	0.4	0.8	1.2	1.6	2.0	2.4	2.8	3.2
*α*																	
-0.5	96.5	95.7	95.3	94.3	92.9	90.8	88.1	84.5	79.4	75.2	71.5	68.3	66.5	65.2	64.3	64.1	62.9
*τ*_1_ = 0.66	(-0.9)	(-1.3)	(-1.6)	(-2.2)	(-2.0)	(-3.1)	(-4.8)	(-6.1)	(-7.5)	(-8.7)	(-8.8)	(-8.1)	(-7.0)	(-7.4)	(-6.4)	(-5.8)	(-5.6)
-0.25	97.4	96.0	94.0	91.2	83.5	73.6	59.2	41.9	26.4	17.2	14.8	16.7	20.4	25.5	29.8	34.7	37.4
*τ*_1_ = 0.58	(1.4)	(1.5)	(1.5)	(1.6)	(0.1)	(-2.5)	(-5.2)	(-8.0)	(-7.8)	(-4.3)	(2.1)	(8.6)	(15.0)	(20.3)	(25.2)	(28.5)	(30.8)
0	97.2	95.3	91.8	84.4	70.2	49.8	25.9	10.0	4.6	10.2	26.6	49.8	71.8	84.6	91.8	95.1	96.9
	(4.5)	(6.2)	(8.5)	(9.4)	(11.2)	(9.9)	(4.2)	(0.5)		(0.7)	(2.7)	(8.2)	(11.5)	(10.7)	(7.6)	(6.4)	(4.4)
0.25	97.0	93.7	88.4	77.1	55.9	30.0	13.1	11.4	26.7	57.6	87.0	97.8	99.8	100.0	100.0	100.0	100.0
*τ*_1_ = 0.41	(9.2)	(14.0)	(20.1)	(25.6)	(25.6)	(17.0)	(8.1)	(0.5)	(-8.3)	(-8.0)	(-2.6)	(0.1)	(0.1)	(0.0)	(0.0)	(0.0)	(0.0)
0.5	96.0	92.4	84.0	68.5	45.3	27.3	24.4	44.5	79.0	96.2	99.9	100.0	100.0	100.0	100.0	100.0	100.0
*τ*_1_ = 0.32	(16.7)	(25.5)	(35.6)	(41.8)	(36.5)	(22.0)	(6.0)	(-8.4)	(-8.1)	(-2.0)	(-0.1)	(0.0)	(0.0)	(0.0)	(0.0)	(0.0)	(0.0)

$\tau_{1}=\tau_{Z=1}=\tau_{0}^{\text{exp}(\alpha )}$/ *τ*_0_ = *τ*_*Z* = 0_ = 0.5

**Table 3:** Bounded cumulative hazard model, exponential censoring, *τ*_0_ = 70%, *p* = 20%, *n*_tot_ = 400, *ξ* = 0.5.

*β*	-3.2	-2.8	-2.4	-2.0	-1.6	-1.2	-0.8	-0.4	0	0.4	0.8	1.2	1.6	2.0	2.4	2.8	3.2
*α*																	
-0.5	69.9	70.7	68.2	67.6	64.4	64.3	61.6	59.5	56.3	55.2	52.9	50.9	49.7	49.8	49.2	49.1	48.9
*τ*_1_ = 0.81	(-7.7)	(-8.5)	(-8.7)	(-8.6)	(-9.5)	(-9.2)	(-10.5)	(-10.4)	(-11.0)	(-10.3)	(-10.5)	(-11.2)	(-10.3)	(-10.2)	(-9.8)	(-10.5)	(-9.7)
-0.25	73.2	69.9	64.1	58.6	52.9	42.8	33.1	23.8	17.2	12.8	10.9	10.4	11.2	12.8	13.5	13.8	15.2
*τ*_1_ = 0.76	(-0.3)	(-1.5)	(-3.1)	(-3.2)	(-3.7)	(-5.3)	(-5.7)	(-7.9)	(-6.1)	(-3.2)	(-0.5)	(1.5)	(4.0)	(6.7)	(7.8)	(8.6)	(10.1)
0	75.0	70.1	62.1	51.4	38.8	26.3	13.8	7.4	4.3	6.1	13.5	25.3	38.8	52.2	64.7	70.1	74.1
	(7.4)	(9.4)	(8.0)	(6.9)	(4.6)	(2.6)	(0.7)	(-0.1)		(-1.0)	(0.9)	(2.3)	(5.0)	(6.4)	(7.9)	(7.5)	(8.0)
0.25	75.4	67.4	58.4	43.9	28.8	16.5	9.2	8.7	17.3	36.3	61.8	81.7	94.1	98.3	99.4	99.6	99.9
*τ*_1_ = 0.63	(18.1)	(16.8)	(18.6)	(16.8)	(13.0)	(9.1)	(4.3)	(0.0)	(-5.8)	(-7.8)	(-7.1)	(-2.9)	(-0.8)	(0.0)	(-0.1)	(0.0)	(0.0)
0.5	73.5	64.6	53.4	37.9	24.3	16.1	17.7	29.5	55.7	81.6	95.9	99.6	100.0	100.0	100.0	100.0	100.0
*τ*_1_ = 0.56	(26.1)	(27.2)	(28.1)	(24.1)	(18.1)	(9.9)	(2.2)	(-6.9)	(-9.6)	(-6.5)	(-1.7)	(-0.1)	(0.0)	(0.0)	(0.0)	(0.0)	(0.0)

$\tau_{1}=\tau_{Z=1}=\tau_{0}^{\text{exp}(\alpha )}$/ *τ*_0_ = *τ*_*Z* = 0_ = 0.7

**Table 4:** Bounded cumulative hazard model, exponential censoring, *τ*_0_ = 30%, *p* = 40%, *n*_tot_ = 400, *ξ* = 0.5.

*β*	-3.2	-2.8	-2.4	-2.0	-1.6	-1.2	-0.8	-0.4	0	0.4	0.8	1.2	1.6	2.0	2.4	2.8	3.2
*α*																	
-0.5	100.0	100.0	100.0	100.0	100.0	100.0	99.9	97.2	80.7	48.5	27.7	27.9	46.5	68.7	83.8	92.6	96.2
*τ*_1_ = 0.48	(0.0)	(0.0)	(0.0)	(0.0)	(0.0)	(0.0)	(-0.1)	(-1.3)	(-7.3)	(-8.5)	(6.3)	(21.9)	(38.0)	(45.3)	(39.6)	(28.4)	(20.0)
-0.25	100.0	100.0	100.0	100.0	100.0	99.8	96.5	70.8	26.7	8.6	20.6	58.5	88.9	98.4	99.9	100.0	100.0
*τ*_1_ = 0.39	(0.0)	(0.0)	(0.0)	(0.0)	(0.0)	(-0.1)	(-1.1)	(-7.1)	(-8.1)	(1.6)	(8.9)	(12.3)	(8.7)	(2.2)	(0.4)	(0.1)	(0.0)
0	100.0	100.0	100.0	100.0	100.0	97.3	72.7	24.0	4.7	22.9	72.2	97.5	99.9	100.0	100.0	100.0	100.0
	(0.0)	(0.0)	(0.0)	(0.0)	(0.1)	(0.2)	(-2.9)	(-2.3)		(-3.9)	(-3.1)	(0.2)	(0.0)	(0.0)	(0.0)	(0.0)	(0.0)
0.25	100.0	100.0	100.0	100.0	98.8	80.7	30.6	6.8	27.1	79.4	98.9	100.0	100.0	100.0	100.0	100.0	100.0
*τ*_1_ = 0.21	(0.0)	(0.0)	(0.0)	(0.0)	(0.3)	(3.8)	(4.1)	(2.0)	(-7.4)	(-6.3)	(-0.4)	(0.0)	(0.0)	(0.0)	(0.0)	(0.0)	(0.0)
0.5	100.0	100.0	100.0	99.5	89.2	43.8	11.5	26.1	79.5	98.9	100.0	100.0	100.0	100.0	100.0	100.0	100.0
*τ*_1_ = 0.14	(0.0)	(0.0)	(0.0)	(0.5)	(4.6)	(10.0)	(6.3)	(-6.1)	(-7.2)	(-0.6)	(0.0)	(0.0)	(0.0)	(0.0)	(0.0)	(0.0)	(0.0)

$\tau_{1}=\tau_{Z=1}=\tau_{0}^{\text{exp}(\alpha )}$/ *τ*_0_ = *τ*_*Z* = 0_ = 0.3

**Table 5:** Bounded cumulative hazard model, exponential censoring, *τ*_0_ = 50%, *p* = 40%, *n*_tot_ = 400, *ξ* = 0.5.

*β*	-3.2	-2.8	-2.4	-2.0	-1.6	-1.2	-0.8	-0.4	0	0.4	0.8	1.2	1.6	2.0	2.4	2.8	3.2
*α*																	
-0.5	100.0	100.0	100.0	100.0	100.0	99.7	97.1	87.6	66.0	41.8	26.8	23.6	27.5	36.3	47.4	56.5	62.1
*τ*_1_ = 0.66	(0.0)	(0.0)	(0.0)	(0.0)	(0.0)	(-0.1)	(-1.1)	(-5.1)	(-10.0)	(-8.4)	(1.2)	(11.1)	(21.9)	(30.3)	(38.1)	(40.6)	(41.1)
-0.25	100.0	100.0	100.0	100.0	99.6	97.0	83.6	51.3	20.9	8.4	12.5	33.0	60.2	82.1	93.4	97.4	98.9
*τ*_1_ = 0.58	(0.0)	(0.0)	(0.0)	(0.0)	(-0.2)	(-0.7)	(-3.6)	(-8.0)	(-6.5)	(0.7)	(6.2)	(11.5)	(15.0)	(11.1)	(7.3)	(3.0)	(1.5)
0	100.0	100.0	100.0	99.7	97.4	84.4	48.6	15.8	4.3	14.8	49.1	84.1	97.7	99.9	100.0	100.0	100.0
	(0.0)	(0.0)	(0.0)	(-0.1)	(0.3)	(0.5)	(-3.4)	(-2.2)		(-2.8)	(-2.5)	(-0.4)	(0.3)	(0.2)	(0.0)	(0.0)	(0.0)
0.25	100.0	100.0	99.9	98.4	88.5	57.6	19.2	6.7	20.6	62.6	93.2	99.4	100.0	100.0	100.0	100.0	100.0
*τ*_1_ = 0.41	(0.0)	(0.0)	(0.0)	(0.5)	(1.9)	(4.8)	(3.0)	(1.0)	(-7.1)	(-7.3)	(-2.7)	(-0.3)	(0.0)	(0.0)	(0.0)	(0.0)	(0.0)
0.5	100.0	99.9	99.2	93.1	66.5	26.9	10.6	22.6	66.2	94.8	99.9	100.0	100.0	100.0	100.0	100.0	100.0
*τ*_1_ = 0.32	(0.0)	(0.1)	(0.4)	(4.4)	(9.1)	(10.0)	(5.7)	(-6.3)	(-10.2)	(-2.3)	(0.0)	(0.0)	(0.0)	(0.0)	(0.0)	(0.0)	(0.0)

$\tau_{1}=\tau_{Z=1}=\tau_{0}^{\text{exp}(\alpha )}$/ *τ*_0_ = *τ*_*Z* = 0_ = 0.5

**Table 6:** Bounded cumulative hazard model, exponential censoring, *τ*_0_ = 70%, *p* = 40%, *n*_tot_ = 400, *ξ* = 0.5.

*β*	-3.2	-2.8	-2.4	-2.0	-1.6	-1.2	-0.8	-0.4	0	0.4	0.8	1.2	1.6	2.0	2.4	2.8	3.2
*α*																	
-0.5	99.9	99.9	99.8	99.1	97.5	91.7	80.5	63.1	43.9	28.8	18.5	16.0	15.1	18.4	21.4	23.4	27.5
*τ*_1_ = 0.81	(0.0)	(-0.1)	(0.1)	(-0.3)	(-0.8)	(-2.6)	(-5.7)	(-9.8)	(-10.9)	(-7.8)	(-3.0)	(3.8)	(7.8)	(12.7)	(16.8)	(18.4)	(20.8)
-0.25	100.0	99.9	99.0	97.1	91.1	76.6	54.5	29.1	13.9	6.7	7.6	16.1	29.9	46.2	60.3	72.4	79.6
*τ*_1_ = 0.76	(0.0)	(0.0)	(-0.2)	(-0.4)	(-1.6)	(-5.6)	(-6.2)	(-8.1)	(-4.6)	(-0.6)	(2.8)	(5.5)	(8.4)	(9.2)	(10.1)	(8.9)	(7.3)
0	99.9	99.5	97.6	92.8	78.4	54.5	26.8	10.1	4.6	10.8	27.0	53.6	78.2	92.0	97.9	99.5	99.9
	(0.1)	(0.3)	(-0.2)	(-0.7)	(-0.6)	(-2.1)	(-3.5)	(-1.7)		(-0.9)	(-2.9)	(-3.8)	(-1.4)	(0.2)	(0.0)	(0.0)	(0.0)
0.25	99.5	98.0	93.8	82.0	60.1	30.0	11.9	6.2	14.1	37.0	71.0	92.6	98.8	99.9	100.0	100.0	100.0
*τ*_1_ = 0.63	(0.1)	(0.3)	(0.1)	(0.7)	(1.6)	(1.8)	(2.0)	(0.4)	(-4.5)	(-9.0)	(-6.8)	(-2.2)	(-0.4)	(-0.1)	(0.0)	(0.0)	(0.0)
0.5	98.8	95.4	86.7	65.7	37.6	14.8	8.3	17.1	45.8	76.4	95.3	99.5	100.0	100.0	100.0	100.0	100.0
*τ*_1_ = 0.56	(0.3)	(0.5)	(3.3)	(4.5)	(6.7)	(5.2)	(2.9)	(-4.2)	(-9.4)	(-7.9)	(-2.3)	(-0.3)	(0.0)	(0.0)	(0.0)	(0.0)	(0.0)

$\tau_{1}=\tau_{Z=1}=\tau_{0}^{\text{exp}(\alpha )}$/ *τ*_0_ = *τ*_*Z* = 0_ = 0.7

For unbalanced designs, *ξ* was set at 0.3 and 0.7. The results are presented in Tables 7–8 and in Tables G-H in S1 File.

**Table 7:** Bounded cumulative hazard model, exponential censoring and unbalanced design, *τ*_0_ = 30%, *p* = 20%, *n*_tot_ = 400, *ξ* = 0.3.

*β*	-3.2	-2.8	-2.4	-2.0	-1.6	-1.2	-0.8	-0.4	0	0.4	0.8	1.2	1.6	2.0	2.4	2.8	3.2
*α*																	
-0.5	100.0	100.0	100.0	100.0	99.9	99.6	97.9	92.0	82.0	69.9	63.6	59.3	58.0	58.4	59.9	61.4	62.3
*τ*_1_ = 0.48	(0.0)	(0.0)	(0.0)	(0.0)	(-0.1)	(-0.1)	(-0.9)	(-3.2)	(-6.9)	(-8.5)	(-5.0)	(0.5)	(8.1)	(13.4)	(18.9)	(24.0)	(25.6)
-0.25	100.0	100.0	100.0	99.9	99.6	96.1	82.8	54.5	30.0	17.4	17.4	24.5	35.1	45.3	53.4	59.0	62.0
*τ*_1_ = 0.39	(0.0)	(0.0)	(0.0)	(0.0)	(0.1)	(0.2)	(-2.4)	(-7.9)	(-8.0)	(-2.1)	(8.7)	(18.5)	(29.5)	(38.5)	(45.1)	(49.0)	(50.8)
0	100.0	100.0	100.0	99.5	95.9	78.0	42.5	13.6	4.6	12.5	34.2	60.4	79.3	88.5	93.8	96.0	97.0
	(0.0)	(0.0)	(0.1)	(0.5)	(2.9)	(6.0)	(4.1)	(1.0)		(1.4)	(6.9)	(15.8)	(17.8)	(15.9)	(14.9)	(12.4)	(11.6)
0.25	100.0	99.9	99.7	97.3	82.4	46.5	15.7	10.4	28.1	61.9	89.1	97.8	99.5	99.9	99.9	100.0	100.0
*τ*_1_ = 0.21	(0.0)	(0.1)	(0.8)	(6.2)	(14.6)	(19.6)	(9.6)	(0.4)	(-7.4)	(-7.3)	(-0.9)	(0.7)	(0.3)	(0.1)	(0.0)	(0.1)	(0.0)
0.5	100.0	99.9	98.2	89.2	58.9	29.1	22.2	46.4	83.2	97.8	99.9	100.0	100.0	100.0	100.0	100.0	100.0
*τ*_1_ = 0.14	(0.0)	(0.6)	(5.8)	(21.7)	(34.2)	(24.5)	(5.8)	(-9.5)	(-6.8)	(-1.0)	(-0.1)	(0.0)	(0.0)	(0.0)	(0.0)	(0.0)	(0.0)

$\tau_{1}=\tau_{Z=1}=\tau_{0}^{\text{exp}(\alpha )}$/ *τ*_0_ = *τ*_*Z* = 0_ = 0.3

**Table 8:** Bounded cumulative hazard model, exponential censoring and unbalanced design, *τ*_0_ = 30%, *p* = 20%, *n*_tot_ = 400, *ξ* = 0.7.

*β*	-3.2	-2.8	-2.4	-2.0	-1.6	-1.2	-0.8	-0.4	0	0.4	0.8	1.2	1.6	2.0	2.4	2.8	3.2
*α*																	
-0.5	92.2	91.9	92.2	91.9	90.8	90.4	88.4	88.6	85.9	84.9	83.0	79.1	76.6	76.8	75.2	73.2	72.9
*τ*_1_ = 0.48	(-3.1)	(-3.0)	(-2.9)	(-3.7)	(-4.1)	(-4.0)	(-5.3)	(-4.6)	(-6.3)	(-6.0)	(-6.3)	(-7.2)	(-7.9)	(-6.7)	(-6.9)	(-6.7)	(-6.9)
-0.25	95.4	94.3	93.3	90.3	84.9	76.5	63.1	46.9	29.6	16.9	13.8	20.5	33.3	49.8	64.5	76.2	82.5
*τ*_1_ = 0.39	(4.3)	(3.9)	(3.4)	(2.7)	(1.9)	(-0.5)	(-4.1)	(-7.6)	(-8.0)	(-4.1)	(4.8)	(15.7)	(26.8)	(35.6)	(38.8)	(37.9)	(32.7)
0	96.6	95.3	93.2	88.9	79.7	60.2	34.0	12.0	4.8	13.1	42.0	77.1	96.1	99.6	99.9	100.0	100.0
	(10.9)	(12.1)	(14.3)	(16.7)	(17.4)	(14.5)	(7.4)	(1.3)		(0.3)	(3.4)	(5.5)	(3.3)	(0.7)	(0.0)	(0.0)	(0.0)
0.25	97.4	95.8	92.3	84.4	68.6	43.1	20.3	13.0	29.3	69.2	94.8	99.6	100.0	100.0	100.0	100.0	100.0
*τ*_1_ = 0.21	(18.7)	(22.1)	(29.3)	(33.2)	(35.3)	(26.7)	(13.6)	(2.1)	(-7.3)	(-6.2)	(-1.7)	(-0.2)	(0.0)	(0.0)	(0.0)	(0.0)	(0.0)
0.5	97.6	94.7	90.6	79.8	60.9	36.4	27.4	46.7	82.1	98.2	100.0	100.0	100.0	100.0	100.0	100.0	100.0
*τ*_1_ = 0.14	(28.2)	(35.0)	(44.5)	(50.6)	(47.2)	(30.7)	(10.1)	(-6.0)	(-6.4)	(-0.7)	(0.0)	(0.0)	(0.0)	(0.0)	(0.0)	(0.0)	(0.0)

$\tau_{1}=\tau_{Z=1}=\tau_{0}^{\text{exp}(\alpha )}$/ *τ*_0_ = *τ*_*Z* = 0_ = 0.3

We also evaluated the impact of a restricted follow-up on the test’s properties by generating censoring times from a uniform distribution lying in a small interval (0, *U*_*max*_), with *U*_*max*_ varying according to the *β* value and set to ensure that 20% of the susceptible patients did not have a sufficient follow-up. The results are reported in Table I (S1 File). For the latter two scenarios, the plateau value was 30% and *p* = 20%.

As stated above, we used a non-parametric maximum likelihood estimator for the cumulative distribution function under the null hypothesis (${\hat{\bar{S}}}_{H_{0}}\left(t\right)$) for the proposed statistic. This estimate was obtained by the pooled-adjacent-violators algorithm available from the ‘isotone’ package [19]. For the classical logrank-type test adapted to interval-censored data, we used the ‘gLRT2’ function from the ‘glrt’ package [20]. The *R*-function for computing the proposed score test is available from the corresponding author on request.

For each scenario, we generated 5,000 datasets. We considered a two-sided test with a 0.05 significance level. The results, in percentages, are summarized in the tables presented below. We report the probabilities of rejecting *H*_0_ for the proposed score test together with the differences observed in statistical power between the score test and the logrank-type test (positive values of differences indicate better power with the proposed score test). The values estimated under the null hypothesis are also reported, highlighted in gray in the tables.

#### Simulation results

For all the situations considered here under the bounded cumulative model (Tables 1–6), the estimated values for the proposed score test under the null hypothesis (*α* = *β* = 0) were within the random sampling fluctuation of the nominal significance level based on the chi-square distribution with two degrees of freedom.

As seen in Tables 1–3 (20% censoring, balanced design), the proposed score test was more powerful than the logrank-type test in most of the scenarios. However, as expected, when *β* = 0, the improper model was a proportional hazard model and the logrank-type test was always more powerful. Table 1 shows that power was up to 53.1% higher for the proposed score test than for the logrank-type test, and never more than 9.6% lower. In Tables 2 and 3, power for the proposed score test was up to 41.8% and 28.1% higher and no more than 8.8% and 11.2% lower, respectively. For any given configuration, power decreased as plateau value increased. This reduced power is not surprising since fewer events are expected with a higher plateau value (as in the configurations in Tables 2 and 3). Moreover, for configurations with low values of *β*, power for the proposed score is lower than for the logrank-type test, as reported above for *β* = 0.

It should be noted that for any given combination of *α* and *β*, the power for the proposed score test is higher than for the logrank-type test when *αβ* > 0; on the other hand, the differences in power between these two score tests are highest in favor of the proposed score test when *αβ* < 0.

These power trends for the proposed score test may be explained by the model from which the test is derived: it supports two parameters for only one covariate. This means that the two parameters compete with each other to some extent. Moreover, a first-order Taylor expansion of *β* around zero gives us: *h*_*Z* = 1_ (*t*) ≈ *h*_*Z* = 0_ (*t*) *e*^*α* + *β*(1 − Λ(*t*))^. We then have a time-dependent hazard ratio. Broadly speaking, at earlier event times (when Λ(*t*) < 1), *α* and *β* each offsets the other when they have opposite signs.

High observed differences in power when *αβ* < 0 can also be explained by the fact that these configurations are clearly not favorable to the logrank-type test because the survival curves cross. For example, when *α* is negative and *β* is positive, the reference group has a lower plateau value and a lower risk of event.

From Tables 4–6, which used 40% censoring, we see the same trends in comparing the power of these two tests. Note that power for the proposed score test compared to that for the logrank-type test was slightly lower than it was in the same scenario with 20% censoring. It was, however, still interesting: up to 45.3% higher and no more than 8.5% lower (*τ*_0_ = 30%). In general, for a given configuration, the power of the proposed score test was higher with a censoring rate of 40% compared to 20%. This increase in power when the censoring rate goes up may be explained by the hazard ratio form when we have a mix of positive and negative contributions associated with *β* and dependent on the follow-up scheme.

Looking at the unbalanced designs (*ξ* = 0.3 and *ξ* = 0.7) in Tables 7–8, we see that the differences in power were quite similar to those observed in the balanced designs. The proposed score test performed better with *ξ* = 0.3 than in situations with *ξ* = 0.7, depending on the sign of *αβ*.

For all the simulated scenarios under a mixture cure model (Tables A-H in S1 File), the estimated values for the proposed score test under the null hypothesis were within the random sampling fluctuation of the nominal significance level. When we look at the power results and differences in power compared with the logrank test, we see that the results were slightly better than those obtained under the cumulative bounded hazard model. As an example, in Table A (S1 File) with *p* = 20%, *τ*_0_ = 30%, *ξ* = 0.5, power for the proposed score test, compared to that with the logrank-type test, was as much as 58.1% higher and no more than 8.7% lower.

In the case where the non-sufficient follow-up condition was violated (Table I in S1 File), the type I error rate was close to the nominal significance level (4.8%). The performances of the proposed score test were slightly lower than that obtained with sufficient follow-up and comparable censoring rates. Moreover, the power gains of the proposed score test compared with the logrank-type test were also lower in this case.

### Testing the influence of gender on ADA occurrence

This dataset came from a cross-sectional study performed by the Immunology Reference Laboratory of Düsseldorf. The study, which was approved by the ethics committee of the medical faculty of the university of Düsseldorf, was part of a databank created by the European ABIRISK consortium [21]. The objective of this consortium is to identify and decipher the impact of bioclinical factors on the immunogenicity of BP across various immune diseases and drugs. In this work, we focused on immunogenicity of interferon products—intramuscular and subcutaneous—in newly treated multiple sclerosis patients. Here, we considered biotherapeutic-naive adult patients whose biological samples were taken within the first 21 months of treatment to be sure to capture potential late events, given that the classical window of appearance for ADAs is 18 months. The dataset comprised patients taking interferon-*β*: 63.3% took interferon-*β*-1a and 36.7% interferon-*β*-1b. Interferon-*β*-1b is only administered subcutaneously whereas interferon-*β*-1a can be administered subcutaneously (55.2%) or intramuscularly (44.8%). We expected a non-negligible proportion of immune-tolerant patients in this cohort. One sample per patient was provided. In all, 969 patients were analyzed, 9.9% of whom had developed ADAs (*n*_*ADA*_ = 96). The sample included 72.6% of women (*n*_*women*_ = 703). The variable Gender was tested.

**Fig 1:**
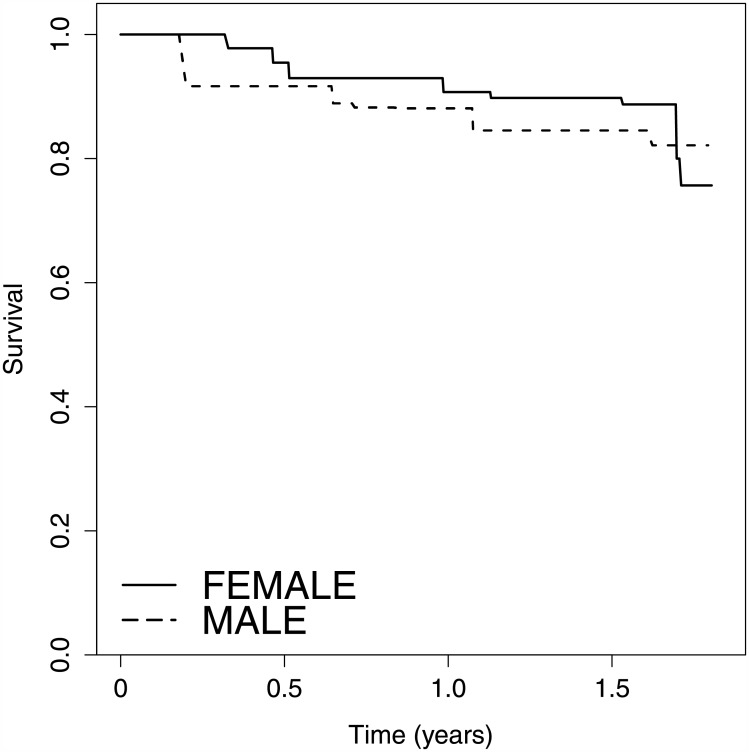
Non-parametric maximum likelihood survival estimate from Dusseldorf data.

Fig 1 displays the non-parametric maximum likelihood estimator of the time-to-ADA survival distribution for each group. The proposed score test produces a significant difference at the global level of 5% with a value of 7.54 (*p*-value = 0.02), whereas the logrank-type test was not significant with a value of 3.33 (*p*-value = 0.07).

## Discussion

Due to time and budget constraints, cross-sectional survival studies often use designs in which patients are randomly sampled at a single point in time. One example is the investigation of BP immunogenicity in a study population expected to include a mixture of immune-tolerant and immune-reactive patients. The analysis of these data requires an extension of classical test statistics for current status data since these statistics are not designed to cope with mixed populations. It is worth noting that this problem is not specific to clinical immunogenicity but is also encountered in oncology (early-stage cancer with cured patients) and infectious diseases (immune individuals, vaccinology), among other fields. In this paper, we proposed a novel two-sample test based on an improper survival model that is well suited for detecting departures from the equality of survival distributions in mixed patient populations. Here, the choice to use a bounded cumulative hazard model was motivated by its interesting mechanistic interpretation of ADA immunogenicity. The proposed score test is designed to detect changes either in the proportion of susceptible patients or in the time-to-event distribution among non-susceptible patients. As seen from our simulations, under the null hypothesis, the proposed test maintains a correct type I error across all the configurations we studied. Looking at the simulation results under the alternative hypotheses, we see that the power of the proposed test is better than that of the logrank-type test in most situations. For a plateau of 30% and a censoring rate of 20%, power increases by up to 53% and decreases to no more than 10% as compared to the logrank-type test. Our test is derived under a model that supports two parameters for only one covariate. Thus, the two parameters compete against each other to some extent. This explains why the power of the proposed score test is lower in situations where there is an opposite directional effect on the time-to-event distribution and the non-susceptible fraction: belonging to group 1, compared to the reference group, entails a lower risk of susceptibility but a higher risk of event for those who are susceptible. The simulation results also show that when censoring is high among the susceptible individuals, the power gains relative to the logrank-type test are preserved. When the sufficient follow-up condition is not verified, that is, when the length of follow-up is shorter than the window of time during which an event can occur, the power of the test is reduced. In that case, its estimated type I error rate is close to the nominal level.

In our immunogenicity study, using our proposed test, we identified a significant association between gender and ADA occurrence. In the analysis considered in this work, an immune-tolerant fraction exists; continuing follow-up enabled many patients to be tested after the first 18 months and therefore allowed an interpretable time sequence for ADA occurrence. We determined that gender plays a role in ADA immunogenicity, a role undetected by the classical logrank-type test but reported in other interferon-*β* cohorts treated for multiple sclerosis [22]. Gender may explain the lack of statistical significance of the logrank-type test, for it mainly acts on the dynamics of ADA production. As seen in Fig 1, men have a higher risk of developing ADA than women and a similar risk of susceptibility.

A limitation of the proposed test is that it can detect only a global difference between groups; it cannot distinguish between a change in the non-susceptible fraction or the survival distribution among the susceptible individuals. Thus, further additional work is needed to derive simple solutions to resolve this problem. Nonetheless, in view of the improved power obtained when the survival distribution changes among susceptible individuals, the use of the proposed score test can be recommended for widespread use when a fraction of non-susceptible individuals is expected. From a practical perspective, it should be borne in mind that this test performs best in situations with a sufficient length of follow-up, that is, a window of observation long enough for the last potential event to occur within it. In other words, we should provide a follow-up adequate for detecting the presence of non-susceptible individuals in the study population. As with the classical logrank-type test, the proposed score test relies upon the assumption that the distribution of the monitoring times is the same across the different groups to be compared. In our study, we can reasonably consider that there is no planned difference in monitoring times between men and women. It should also be noted that the proposed test can be extended to take other factors into account, by developing a stratified version with strata defined by the levels of the factors. Finally, we think that our proposed test is both easy to implement and valuable in cross-sectional survival studies with mixed populations, given that the logrank-type test can be ineffective in this situation.

## Appendix

The logrank-type statistic corresponds to the grouped likelihood score test for interval-censored data deduced under proportional hazards alternatives [18]. This is the most commonly used semi-parametric test adapted to interval-censored data.

With current status data, the simplified log-likelihood is:

$$LLik\;(\beta ,S\;(.))=\sum_{i=1}^{n}\;\left(1-\delta_{i}\right)\log[S\;(c_{i}|z_{i})]+\delta_{i}\log[1-S\;(c_{i}|z_{i})]$$

Thus, the corresponding score statistic, denoted by $U_{0}^{cs}$, is:

$$\begin{array}{c} U_{0}^{cs}=\sum_{i=1}^{n}\;z_{i}\frac{\log\hat{S}\left(C_{i}\right)}{1-\hat{S}\left(C_{i}\right)}\;[1-\hat{S}\left(C_{i}\right)-\delta_{i}]\; \end{array}$$

In the logrank-type test used in the simulations, the covariance matrix is derived under the null hypothesis, see Sun [23] for details. We chose the function ‘gLRT2’ of the package ‘glrt’ for calculation speed considerations. Other procedures (exact or asymptotic permutation methods, score test with the observed Fisher’s information and multiple imputations) are available in different R packages such as ‘interval’ [24] and ‘FHtest’ [25].

## Supporting information

S1 File Supplementary tables. **Table A,** Mixture cure model, exponential censoring, *τ*_0_ = 30%, *p* = 20%, *n*_tot_ = 400, *ξ* = 0.5. **Table B,** Mixture cure model, exponential censoring, *τ*_0_ = 50%, *p* = 20%, *n*_tot_ = 400, *ξ* = 0.5. **Table C,** Mixture cure model, exponential censoring, *τ*_0_ = 70%, *p* = 20%, *n*_tot_ = 400, *ξ* = 0.5. **Table D,** Mixture cure model, exponential censoring, *τ*_0_ = 30%, *p* = 40%, *n*_tot_ = 400, *ξ* = 0.5. **Table E,** Mixture cure model, exponential censoring, *τ*_0_ = 50%, *p* = 40%, *n*_tot_ = 400, *ξ* = 0.5. **Table F,** Mixture cure model, exponential censoring, *τ*_0_ = 70%, *p* = 40%, *n*_tot_ = 400, *ξ* = 0.5. **Table G,** Mixture cure model, exponential censoring, *τ*_0_ = 30%, *p* = 20%, *n*_tot_ = 400, *ξ* = 0.3. **Table H,** Mixture cure model, exponential censoring, *τ*_0_ = 30%, *p* = 20%, *n*_tot_ = 400, *ξ* = 0.7. **Table I,** Bounded cumulative hazard model, uniform censoring and insufficient follow-up, *τ*_0_ = 30%, *n*_tot_ = 400, *ξ* = 0.5.
(PDF)
