# Supplementary material for: A score test for comparing cross-sectional survival data with a fraction of non-susceptible patients and its application in clinical immunology
Source: PLoS One. 2017 Jun 30;12(6):e0179896. doi: 10.1371/journal.pone.0179896 (PMC5493340; doi:10.1371/journal.pone.0179896)
Supplement: S1 File — Table A, Mixture cure model, exponential censoring, τ0 = 30%, p = 20%, ntot = 400, ξ = 0.5. Table B, Mixture cure model, exponential censoring, τ0 = 50%, p = 20%, ntot = 400, ξ = 0.5. Table C, Mixture cure model, exponential censoring, τ0 = 70%, p = 20%, ntot = 400, ξ = 0.5. Table D, Mixture cure model, exponential censoring, τ0 = 30%, p = 40%, ntot = 400, ξ = 0.5. Table E, Mixture cure model, exponential censoring, τ0 = 50%, p = 40%, ntot = 400, ξ = 0.5. Table F, Mixture cure model, exponential censoring, τ0 = 70%, p = 40%, ntot = 400, ξ = 0.5. Table G, Mixture cure model, exponential censoring, τ0 = 30%, p = 20%, ntot = 400, ξ = 0.3. Table H, Mixture cure model, exponential censoring, τ0 = 30%, p = 20%, ntot = 400, ξ = 0.7. Table I, Bounded cumulative hazard model, uniform censoring and insufficient follow-up, τ0 = 30%, ntot = 400, ξ = 0.5. (PDF) [file pone.0179896.s001.pdf]

Supplementary materials

Table A. Mixture cure model, exponential censoring, $\tau_0 = 30\%$, $p = 20\%$, $n_{\text{tot}} = 400$, $\xi = 0.5$.

α \ β	-3.2	-2.8	-2.4	-2.0	-1.6	-1.2	-0.8	-0.4	0	0.4	0.8	1.2	1.6	2.0	2.4	2.8	3.2
-0.5 ($\tau_1 = 0.48$)	100.0 (0.0)	100.0 (0.1)	100.0 (0.1)	99.9 (0.0)	99.6 (0.0)	98.7 (-0.6)	96.9 (-1.3)	94.0 (-2.5)	88.8 (-4.8)	83.0 (-6.1)	77.7 (-4.8)	75.7 (-0.2)	73.6 (3.1)	72.4 (8.2)	72.2 (11.5)	73.2 (14.7)	73.0 (17.1)
-0.25 ($\tau_1 = 0.39$)	100.0 (0.1)	100.0 (0.3)	99.9 (0.2)	99.5 (0.9)	98.4 (1.4)	93.8 (0.9)	79.1 (-2.4)	53.8 (-8.7)	30.7 (-8.3)	20.4 (0.1)	22.7 (14.0)	32.4 (27.2)	48.9 (41.6)	62.1 (52.0)	71.4 (57.1)	77.1 (58.1)	82.0 (58.1)
0	100.0 (0.4)	100.0 (0.8)	99.8 (1.4)	98.8 (4.2)	95.0 (9.7)	82.0 (14.3)	49.3 (10.0)	15.6 (2.3)	4.8	15.5 (2.2)	48.4 (10.6)	80.2 (13.6)	95.2 (9.1)	99.1 (3.9)	99.9 (1.5)	100.0 (1.0)	100.0 (0.3)
0.25 ($\tau_1 = 0.21$)	100.0 (0.8)	99.9 (2.0)	99.7 (4.6)	98.4 (12.8)	92.9 (28.7)	69.8 (38.1)	34.1 (25.5)	14.0 (6.8)	24.7 (-7.6)	66.0 (-8.0)	94.7 (-0.9)	99.8 (0.1)	100.0 (0.0)	100.0 (0.0)	100.0 (0.0)	100.0 (0.0)	100.0 (0.0)
0.5 ($\tau_1 = 0.14$)	100.0 (1.4)	99.9 (3.8)	99.7 (10.4)	98.2 (25.1)	90.4 (49.2)	68.7 (57.0)	41.9 (35.8)	38.9 (8.1)	71.8 (-7.5)	96.4 (-1.9)	99.9 (-0.1)	100.0 (0.0)	100.0 (0.0)	100.0 (0.0)	100.0 (0.0)	100.0 (0.0)	100.0 (0.0)

$\tau_1 = \tau_{Z=1} = \tau_0^{\exp(\alpha)}$ / $\tau_0 = \tau_{Z=0} = 0.3$

Table B. Mixture cure model, exponential censoring, $\tau_0 = 50\%$, $p = 20\%$, $n_{\text{tot}} = 400$, $\xi = 0.5$.

α \ β	-3.2	-2.8	-2.4	-2.0	-1.6	-1.2	-0.8	-0.4	0	0.4	0.8	1.2	1.6	2.0	2.4	2.8	3.2
-0.5 ($\tau_1 = 0.66$)	96.2 (-1.3)	96.3 (-0.8)	95.5 (-1.1)	94.0 (-1.9)	92.8 (-2.4)	90.5 (-3.3)	86.1 (-5.6)	82.3 (-7.0)	78.8 (-7.9)	73.6 (-8.3)	70.8 (-8.0)	68.9 (-7.9)	66.7 (-7.6)	65.4 (-6.3)	65.9 (-4.7)	63.7 (-5.4)	64.4 (-4.7)
-0.25 ($\tau_1 = 0.58$)	97.8 (1.9)	95.7 (1.4)	94.1 (1.3)	90.4 (2.0)	83.7 (0.7)	72.6 (-1.8)	56.9 (-5.1)	39.0 (-8.8)	24.3 (-7.7)	17.6 (-3.2)	15.8 (3.1)	18.1 (10.4)	24.0 (18.2)	27.8 (22.5)	33.8 (28.2)	37.8 (31.9)	39.9 (33.6)
0	97.6 (5.4)	96.6 (6.3)	93.4 (9.7)	86.0 (11.9)	73.9 (12.7)	52.4 (10.4)	27.7 (4.8)	10.0 (0.6)	4.6	9.5 (0.5)	27.7 (5.5)	51.9 (11.0)	73.5 (12.2)	86.8 (10.9)	93.0 (9.2)	95.7 (6.5)	97.3 (4.6)
0.25 ($\tau_1 = 0.41$)	97.6 (8.7)	96.6 (13.6)	92.3 (19.9)	82.9 (26.9)	66.5 (31.2)	39.5 (25.1)	17.2 (11.7)	11.4 (1.9)	24.0 (-6.2)	53.5 (-8.5)	84.4 (-2.2)	97.2 (0.1)	99.6 (-0.1)	100.0 (0.1)	100.0 (0.0)	100.0 (0.0)	100.0 (0.0)
0.5 ($\tau_1 = 0.32$)	97.8 (14.7)	96.0 (21.9)	90.5 (33.7)	79.8 (43.6)	62.2 (46.5)	38.9 (33.8)	27.6 (16.0)	36.4 (-3.3)	67.5 (-9.8)	93.6 (-2.8)	99.6 (-0.2)	100.0 (0.0)	100.0 (0.0)	100.0 (0.0)	100.0 (0.0)	100.0 (0.0)	100.0 (0.0)

$\tau_1 = \tau_{Z=1} = \tau_0^{\exp(\alpha)}$ / $\tau_0 = \tau_{Z=0} = 0.5$

Table C. Mixture cure model, exponential censoring, $\tau_0 = 70\%$, $p = 20\%$, $n_{\text{tot}} = 400$, $\xi = 0.5$.

α \ β	-3.2	-2.8	-2.4	-2.0	-1.6	-1.2	-0.8	-0.4	0	0.4	0.8	1.2	1.6	2.0	2.4	2.8	3.2
-0.5 ($\tau_1 = 0.81$)	70.4 (-8.0)	69.5 (-8.6)	69.2 (-8.1)	67.7 (-8.9)	64.9 (-8.3)	63.6 (-9.2)	61.3 (-10.2)	59.1 (-10.9)	55.8 (-11.3)	53.9 (-9.2)	51.2 (-10.9)	49.9 (-10.5)	50.8 (-10.0)	48.7 (-10.2)	48.1 (-9.7)	47.9 (-10.0)	47.4 (-10.2)
-0.25 ($\tau_1 = 0.76$)	72.6 (-0.2)	70.1 (-0.6)	66.4 (-0.8)	58.7 (-2.9)	52.8 (-3.7)	41.6 (-5.7)	30.9 (-7.4)	23.7 (-6.3)	16.4 (-5.6)	13.4 (-2.8)	10.8 (-0.5)	10.4 (1.6)	12.0 (5.2)	13.4 (7.5)	13.8 (9.0)	16.0 (10.5)	15.6 (10.8)
0	75.5 (9.4)	70.7 (8.8)	62.9 (7.2)	52.8 (8.3)	39.4 (5.9)	25.7 (3.6)	14.2 (0.2)	7.4 (-0.4)	4.7	7.3 (0.2)	14.5 (1.0)	27.4 (3.5)	40.0 (5.7)	53.7 (7.8)	63.3 (8.8)	71.5 (10.6)	75.2 (8.3)
0.25 ($\tau_1 = 0.63$)	77.2 (17.9)	69.3 (18.2)	60.4 (19.2)	48.8 (18.7)	33.3 (16.3)	18.8 (9.5)	9.9 (5.2)	8.4 (-0.7)	15.3 (-4.7)	35.1 (-8.4)	60.4 (-5.8)	81.4 (-2.6)	93.5 (-0.7)	98.3 (0.2)	99.5 (0.0)	99.9 (0.1)	100.0 (0.1)
0.5 ($\tau_1 = 0.56$)	78.6 (27.0)	69.7 (27.9)	58.4 (30.0)	45.7 (29.6)	30.6 (23.4)	19.5 (14.1)	17.1 (5.0)	27.3 (-5.7)	51.1 (-10.6)	77.9 (-7.7)	94.5 (-2.5)	99.4 (-0.3)	100.0 (0.0)	100.0 (0.0)	100.0 (0.0)	100.0 (0.0)	100.0 (0.0)

$\tau_1 = \tau_{Z=1} = \tau_0^{\exp(\alpha)}$ / $\tau_0 = \tau_{Z=0} = 0.7$

Table D. Mixture cure model, exponential censoring, $\tau_0 = 30\%$, $p = 40\%$, $n_{\text{tot}} = 400$, $\xi = 0.5$.

$\boldsymbol{\alpha}$ \ $\boldsymbol{\beta}$	-3.2	-2.8	-2.4	-2.0	-1.6	-1.2	-0.8	-0.4	0	0.4	0.8	1.2	1.6	2.0	2.4	2.8	3.2
-0.5	100.0	100.0	100.0	100.0	100.0	100.0	99.6	94.2	69.1	38.9	30.1	42.1	62.6	81.1	91.6	96.5	97.9
$\tau_1 = 0.48$	(0.0)	(0.0)	(0.0)	(0.0)	(0.0)	(0.0)	(-0.2)	(-2.6)	(-9.5)	(-2.6)	(17.6)	(37.1)	(49.1)	(47.5)	(36.5)	(26.1)	(17.4)
-0.25	100.0	100.0	100.0	100.0	100.0	99.8	95.0	65.0	20.0	9.5	34.2	75.2	96.0	99.6	100.0	100.0	100.0
$\tau_1 = 0.39$	(0.0)	(0.0)	(0.0)	(0.0)	(0.0)	(0.0)	(-1.4)	(-7.8)	(-6.9)	(4.3)	(15.2)	(15.6)	(7.4)	(1.6)	(0.1)	(0.0)	(0.0)
0	100.0	100.0	100.0	100.0	100.0	98.5	77.8	25.2	4.5	26.5	78.6	98.6	99.9	100.0	100.0	100.0	100.0
	(0.0)	(0.0)	(0.0)	(0.0)	(0.1)	(0.4)	(-0.7)	(-3.2)		(-2.0)	(0.0)	(-0.1)	(0.0)	(0.0)	(0.0)	(0.0)	(0.0)
0.25	100.0	100.0	100.0	100.0	99.9	93.5	55.2	10.5	16.8	69.8	98.1	100.0	100.0	100.0	100.0	100.0	100.0
$\tau_1 = 0.21$	(0.0)	(0.0)	(0.0)	(0.0)	(0.3)	(3.0)	(6.6)	(4.4)	(-4.2)	(-8.1)	(-0.6)	(0.0)	(0.0)	(0.0)	(0.0)	(0.0)	(0.0)
0.5	100.0	100.0	100.0	100.0	99.5	87.0	38.4	13.0	46.1	93.1	99.9	100.0	100.0	100.0	100.0	100.0	100.0
$\tau_1 = 0.14$	(0.0)	(0.0)	(0.0)	(0.0)	(1.0)	(9.7)	(16.1)	(6.4)	(-9.3)	(-3.3)	(-0.1)	(0.0)	(0.0)	(0.0)	(0.0)	(0.0)	(0.0)

$\tau_1 = \tau_{Z=1} = \tau_0^{\exp(\alpha)}$ / $\tau_0 = \tau_{Z=0} = 0.3$

Table E. Mixture cure model, exponential censoring, $\tau_0 = 50\%$, $p = 40\%$, $n_{\text{tot}} = 400$, $\xi = 0.5$.

$\boldsymbol{\alpha}$ \ $\boldsymbol{\beta}$	-3.2	-2.8	-2.4	-2.0	-1.6	-1.2	-0.8	-0.4	0	0.4	0.8	1.2	1.6	2.0	2.4	2.8	3.2
-0.5	100.0	100.0	100.0	100.0	99.9	99.6	96.3	85.4	61.9	37.8	25.5	26.1	33.1	42.9	52.5	61.8	68.8
$\tau_1 = 0.66$	(0.0)	(0.0)	(0.0)	(0.0)	(-0.1)	(-0.1)	(-1.7)	(-5.5)	(-10.2)	(-7.0)	(4.5)	(17.4)	(27.7)	(36.0)	(40.8)	(44.7)	(45.7)
-0.25	100.0	100.0	100.0	100.0	99.6	96.5	82.2	48.5	17.9	8.1	15.5	39.8	69.7	87.0	95.0	98.7	99.5
$\tau_1 = 0.58$	(0.0)	(0.0)	(0.0)	(0.1)	(0.0)	(-0.7)	(-4.3)	(-8.9)	(-5.3)	(1.8)	(8.4)	(15.5)	(17.1)	(12.0)	(6.3)	(2.8)	(1.4)
0	100.0	100.0	100.0	99.9	98.0	87.1	53.4	17.6	4.6	16.4	52.5	87.2	98.1	99.8	100.0	100.0	100.0
	(0.0)	(0.0)	(0.0)	(0.0)	(0.0)	(1.1)	(-1.6)	(-1.5)		(-2.3)	(-1.4)	(0.1)	(0.4)	(0.0)	(0.0)	(0.0)	(0.0)
0.25	100.0	100.0	100.0	99.3	93.8	68.3	26.8	7.7	16.5	55.8	91.7	99.6	100.0	100.0	100.0	100.0	100.0
$\tau_1 = 0.41$	(0.0)	(0.0)	(0.1)	(0.3)	(3.0)	(4.0)	(4.7)	(2.3)	(-4.3)	(-8.8)	(-2.9)	(-0.1)	(0.0)	(0.0)	(0.0)	(0.0)	(0.0)
0.5	100.0	100.0	99.9	98.6	85.3	50.9	17.6	13.9	49.0	88.8	99.5	100.0	100.0	100.0	100.0	100.0	100.0
$\tau_1 = 0.32$	(0.0)	(0.0)	(0.4)	(2.5)	(8.8)	(14.6)	(10.6)	(-0.5)	(-10.6)	(-4.5)	(-0.4)	(0.0)	(0.0)	(0.0)	(0.0)	(0.0)	(0.0)

$\tau_1 = \tau_{Z=1} = \tau_0^{\exp(\alpha)}$ / $\tau_0 = \tau_{Z=0} = 0.5$

Table F. Mixture cure model, exponential censoring, $\tau_0 = 70\%$, $p = 40\%$, $n_{\text{tot}} = 400$, $\xi = 0.5$.

$\boldsymbol{\alpha}$ \ $\boldsymbol{\beta}$	-3.2	-2.8	-2.4	-2.0	-1.6	-1.2	-0.8	-0.4	0	0.4	0.8	1.2	1.6	2.0	2.4	2.8	3.2
-0.5	100.0	100.0	99.7	98.9	96.9	91.3	79.8	61.9	41.2	27.4	19.1	15.4	16.5	19.4	24.0	25.5	28.6
$\tau_1 = 0.81$	(0.0)	(0.0)	(-0.1)	(-0.4)	(-0.8)	(-2.6)	(-6.0)	(-9.9)	(-11.3)	(-6.6)	(-1.3)	(4.0)	(9.8)	(14.2)	(18.4)	(20.3)	(22.0)
-0.25	99.9	99.9	99.3	97.4	91.1	76.7	54.5	29.8	12.4	6.8	8.3	18.2	32.0	48.6	64.0	72.9	80.8
$\tau_1 = 0.76$	(0.0)	(0.0)	(0.0)	(-0.3)	(-1.5)	(-4.6)	(-7.2)	(-7.3)	(-4.4)	(0.2)	(3.1)	(7.1)	(10.2)	(10.8)	(10.6)	(8.5)	(7.5)
0	99.9	99.5	98.4	93.0	81.4	55.1	28.0	10.2	4.9	10.4	28.5	56.2	80.6	92.7	98.3	99.6	99.8
	(0.0)	(-0.1)	(0.1)	(0.1)	(-0.2)	(-3.0)	(-2.3)	(-1.2)		(-1.2)	(-2.3)	(-2.4)	(-0.3)	(0.0)	(0.3)	(0.1)	(-0.1)
0.25	99.8	98.9	95.9	85.4	65.2	35.5	13.3	6.3	12.2	36.7	70.0	91.6	98.6	99.9	100.0	100.0	100.0
$\tau_1 = 0.63$	(0.0)	(0.2)	(0.5)	(1.4)	(3.2)	(3.0)	(2.2)	(0.6)	(-4.1)	(-8.9)	(-7.0)	(-2.6)	(-0.4)	(0.0)	(0.0)	(0.0)	(0.0)
0.5	99.2	97.8	91.5	75.2	48.6	21.4	8.7	13.3	37.2	71.7	94.3	99.5	100.0	100.0	100.0	100.0	100.0
$\tau_1 = 0.56$	(0.0)	(0.5)	(2.8)	(6.0)	(8.5)	(7.1)	(3.5)	(-1.8)	(-10.5)	(-8.6)	(-2.7)	(-0.2)	(0.0)	(0.0)	(0.0)	(0.0)	(0.0)

$\tau_1 = \tau_{Z=1} = \tau_0^{\exp(\alpha)}$ / $\tau_0 = \tau_{Z=0} = 0.7$

Table G. Mixture cure model, exponential censoring, $\tau_0 = 30\%$, $p = 20\%$, $n_{\text{tot}} = 400$, $\xi = 0.3$.

α \ β	-3.2	-2.8	-2.4	-2.0	-1.6	-1.2	-0.8	-0.4	0	0.4	0.8	1.2	1.6	2.0	2.4	2.8	3.2
-0.5	100.0	100.0	100.0	100.0	99.9	99.5	96.6	90.5	79.1	69.0	63.3	62.3	62.3	63.9	64.3	65.0	66.3
$\tau_1 = 0.48$	(0.0)	(0.0)	(0.0)	(0.0)	(0.0)	(-0.1)	(-1.6)	(-4.2)	(-7.4)	(-7.1)	(-3.0)	(5.1)	(12.8)	(19.1)	(23.9)	(27.6)	(28.7)
-0.25	100.0	100.0	100.0	99.9	99.5	95.3	79.1	50.2	26.9	17.3	22.1	32.2	43.4	53.3	60.5	65.1	69.1
$\tau_1 = 0.39$	(0.0)	(0.0)	(0.0)	(0.0)	(0.4)	(0.3)	(-3.3)	(-7.5)	(-6.5)	(1.3)	(13.1)	(27.2)	(38.0)	(46.0)	(52.7)	(55.0)	(57.2)
0	100.0	100.0	100.0	99.8	97.0	83.0	46.1	14.2	5.4	13.5	37.8	65.8	83.0	91.3	94.7	96.1	97.3
	(0.0)	(0.0)	(0.0)	(0.4)	(3.1)	(8.0)	(6.5)	(1.2)		(2.1)	(11.2)	(19.3)	(21.9)	(19.2)	(16.1)	(14.5)	(11.9)
0.25	100.0	100.0	100.0	99.4	93.6	67.7	28.8	10.6	20.4	56.1	86.5	97.4	99.5	100.0	100.0	100.0	100.0
$\tau_1 = 0.21$	(0.0)	(0.0)	(0.3)	(2.5)	(14.4)	(26.6)	(18.9)	(4.7)	(-6.8)	(-7.5)	(-1.1)	(0.9)	(0.7)	(0.4)	(0.1)	(0.1)	(0.0)
0.5	100.0	100.0	99.9	98.9	89.6	60.8	31.4	32.0	64.5	92.5	99.5	100.0	100.0	100.0	100.0	100.0	100.0
$\tau_1 = 0.14$	(0.0)	(0.0)	(0.8)	(7.3)	(31.2)	(45.4)	(26.9)	(4.1)	(-11.0)	(-3.6)	(-0.2)	(0.0)	(0.0)	(0.0)	(0.0)	(0.0)	(0.0)

$\tau_1 = \tau_{Z=1} = \tau_0^{\exp(\alpha)} / \tau_0 = \tau_{Z=0} = 0.3$

Table H. Mixture cure model, exponential censoring, $\tau_0 = 30\%$, $p = 20\%$, $n_{\text{tot}} = 400$, $\xi = 0.7$.

α \ β	-3.2	-2.8	-2.4	-2.0	-1.6	-1.2	-0.8	-0.4	0	0.4	0.8	1.2	1.6	2.0	2.4	2.8	3.2
-0.5	92.7	91.5	91.8	91.8	91.6	90.2	88.7	87.5	85.6	83.2	81.0	78.6	77.4	74.9	74.2	73.7	73.7
$\tau_1 = 0.48$	(-2.7)	(-3.4)	(-2.8)	(-3.1)	(-3.3)	(-4.0)	(-5.1)	(-5.0)	(-5.9)	(-7.1)	(-7.7)	(-7.3)	(-6.3)	(-7.4)	(-6.6)	(-5.7)	(-5.4)
-0.25	95.3	95.2	93.2	90.8	86.4	77.8	63.2	42.5	24.5	16.9	17.1	26.3	41.7	59.9	72.8	81.6	87.1
$\tau_1 = 0.39$	(4.2)	(5.8)	(3.7)	(4.1)	(3.2)	(0.6)	(-2.5)	(-9.9)	(-8.3)	(-2.7)	(9.0)	(21.3)	(34.0)	(42.8)	(44.1)	(40.6)	(36.8)
0	97.5	95.9	94.8	90.5	81.6	63.6	38.5	14.0	4.6	13.6	45.5	82.7	97.4	99.8	100.0	100.0	100.0
	(11.4)	(12.6)	(16.7)	(18.4)	(18.2)	(17.5)	(10.6)	(1.9)		(0.4)	(6.0)	(8.5)	(3.2)	(0.5)	(0.0)	(0.0)	(0.0)
0.25	98.4	97.4	96.3	91.7	81.9	60.3	31.7	13.9	21.8	60.5	93.7	99.9	100.0	100.0	100.0	100.0	100.0
$\tau_1 = 0.21$	(19.8)	(23.8)	(28.7)	(36.3)	(42.7)	(38.9)	(23.7)	(6.5)	(-7.1)	(-7.4)	(-0.9)	(0.1)	(0.0)	(0.0)	(0.0)	(0.0)	(0.0)
0.5	98.9	98.3	96.3	91.7	83.0	61.7	40.1	35.4	62.0	93.1	99.8	100.0	100.0	100.0	100.0	100.0	100.0
$\tau_1 = 0.14$	(26.8)	(34.7)	(41.6)	(51.9)	(61.0)	(53.4)	(32.5)	(6.8)	(-7.9)	(-3.2)	(-0.1)	(0.0)	(0.0)	(0.0)	(0.0)	(0.0)	(0.0)

$\tau_1 = \tau_{Z=1} = \tau_0^{\exp(\alpha)} / \tau_0 = \tau_{Z=0} = 0.3$

Table I. Bounded cumulative hazard model, uniform censoring and insufficient follow-up, $\tau_0 = 30\%$, $n_{\text{tot}} = 400$, $\xi = 0.5$.

α \ β	-3.2	-2.8	-2.4	-2.0	-1.6	-1.2	-0.8	-0.4	0	0.4	0.8	1.2	1.6	2.0	2.4	2.8	3.2
-0.5	100.0	100.0	100.0	100.0	100.0	100.0	100.0	98.3	75.4	26.5	7.5	19.2	51.7	79.6	90.7	96.6	98.4
$\tau_1 = 0.48$	(0.0)	(0.0)	(0.0)	(0.0)	(0.0)	(0.0)	(0.0)	(-0.9)	(-8.0)	(-6.3)	(1.6)	(4.5)	(5.1)	(5.8)	(4.6)	(3.3)	(2.1)
-0.25	100.0	100.0	100.0	100.0	100.0	100.0	98.5	78.8	24.5	5.3	23.4	68.7	93.8	99.1	99.9	100.0	100.0
$\tau_1 = 0.39$	(0.0)	(0.0)	(0.0)	(0.0)	(0.0)	(0.0)	(-0.8)	(-6.2)	(-8.4)	(0.3)	(-2.2)	(-3.1)	(-1.0)	(0.0)	(0.1)	(0.0)	(0.0)
0	100.0	100.0	100.0	100.0	100.0	98.2	78.7	26.8	4.8	25.7	79.6	98.4	99.9	100.0	100.0	100.0	100.0
	(0.0)	(0.0)	(0.0)	(0.0)	(0.0)	(-0.7)	(-6.1)	(-7.3)		(-6.9)	(-5.8)	(-0.5)	(0.0)	(0.0)	(0.0)	(0.0)	(0.0)
0.25	100.0	100.0	100.0	99.7	96.9	76.4	28.4	5.8	28.6	85.1	99.4	100.0	100.0	100.0	100.0	100.0	100.0
$\tau_1 = 0.21$	(0.0)	(0.0)	(0.1)	(0.1)	(-0.3)	(-2.2)	(-1.1)	(0.8)	(-8.7)	(-5.2)	(-0.4)	(0.0)	(0.0)	(0.0)	(0.0)	(0.0)	(0.0)
0.5	99.9	99.7	98.5	93.5	70.9	28.7	8.9	33.6	87.8	99.7	100.0	100.0	100.0	100.0	100.0	100.0	100.0
$\tau_1 = 0.14$	(0.6)	(1.0)	(2.3)	(6.1)	(9.0)	(7.8)	(3.3)	(-7.8)	(-5.1)	(-0.2)	(0.0)	(0.0)	(0.0)	(0.0)	(0.0)	(0.0)	(0.0)

$\tau_1 = \tau_{Z=1} = \tau_0^{\exp(\alpha)} / \tau_0 = \tau_{Z=0} = 0.3$
